# Propranolol Reduces Parkinson's Tremor and Inhibits Tremor‐Related Activity in the Motor Cortex: A Placebo‐Controlled Crossover Trial

**DOI:** 10.1002/ana.27159

**Published:** 2024-12-21

**Authors:** Anouk van der Heide, Maaike Wessel, Danae Papadopetraki, Dirk E.M. Geurts, Teije H. van Prooije, Frank Gommans, Bastiaan R. Bloem, Michiel F. Dirkx, Rick C. Helmich

**Affiliations:** ^1^ Department of Neurology, Centre of Expertise for Parkinson and Movement Disorders Radboud University Medical Centre Nijmegen The Netherlands; ^2^ Donders Institute for Brain, Cognition, and Behavior, Centre for Cognitive Neuroimaging Radboud University Nijmegen Nijmegen The Netherlands; ^3^ Department of Psychiatry Radboud University Medical Centre Nijmegen The Netherlands; ^4^ Department of Cardiology, Maxima Medical Centre Veldhoven The Netherlands

## Abstract

**Objective:**

Parkinson's disease (PD) resting tremor is thought to be initiated in the basal ganglia and amplified in the cerebello‐thalamo‐cortical circuit. Because stress worsens tremor, the noradrenergic system may play a role in amplifying tremor. We tested if and how propranolol, a non‐selective beta‐adrenergic receptor antagonist, reduces PD tremor and whether or not this effect is specific to stressful conditions.

**Methods:**

In a cross‐over, double‐blind intervention study, participants with PD resting tremor received propranolol (40 mg, single dose) or placebo (counter‐balanced) on 2 different days. During both days, we assessed tremor severity (with accelerometry) and tremor‐related brain activity (with functional magnetic resonance imaging), as well as heart rate and pupil diameter, while subjects performed a stressful cognitive load task that has been linked to the noradrenergic system. We tested for effects of drug (propranolol vs placebo) and stress (cognitive load vs rest) on tremor power and tremor‐related brain activity.

**Results:**

We included 27 PD patients with prominent resting tremor. Tremor power significantly increased during cognitive load versus rest (*F*[1,19] = 13.8; *p* = 0.001; $\eta_{p}^{2}$ = 0.42) and decreased by propranolol versus placebo (*F*[1,19] = 6.4; *p* = 0.02; $\eta_{p}^{2}$ = 0.25), but there was no interaction. We observed task‐related brain activity in a stress‐sensitive cognitive control network and tremor power‐related activity in the cerebello‐thalamo‐cortical circuit. Propranolol significantly reduced tremor‐related activity in the motor cortex compared to placebo (*F*[1,21] = 5.3; *p* = 0.03; $\eta_{p}^{2}$ = 0.20), irrespective of cognitive load.

**Interpretation:**

Our findings indicate that propranolol has a general, context‐independent, tremor‐reducing effect that may be implemented at the level of the primary motor cortex. ANN NEUROL 2025;97:741–752

Parkinson's disease (PD) is characterized by progressive degeneration of the main ascending neurotransmitter systems, including the dopaminergic, noradrenergic, and serotonergic systems.^1^, ^2^ Although nigro‐striatal dopamine depletion is related to bradykinesia and rigidity, this association is less clear for resting tremor.^3^, ^4^, ^5^ Clinical evidence suggests an additional role for the noradrenergic system in the pathophysiology of PD tremor. Specifically, PD tremor increases during experimentally induced stress, such as cognitively demanding tasks,^6^, ^7^ and patients rate tremor as their most stress‐sensitive symptom.^8^ Furthermore, although levodopa is effective in reducing tremor,^9^, ^10^, ^11^ this effect is diminished by stressful contexts.^12^ This highlights an important clinical problem that people experience most tremor during psychological stress, when dopaminergic treatment is least effective. These observations suggest that the noradrenergic system is involved in the pathophysiology of PD tremor, but the mechanisms are unclear.

According to the “dimmer‐switch hypothesis” of PD tremor, pathological activity in the striato‐pallidal circuit triggers tremor onset analogous to a light switch,^13^ whereas increased activity in the cerebello‐thalamo‐cortical circuit amplifies tremor power analogous to a light dimmer. We have previously shown that cognitive load, which activates the noradrenergic system, increases PD tremor through excitatory influences on the thalamus.^6^ Other findings suggest that the involvement of the noradrenergic system in PD tremor may not be restricted to stressful situations, and spontaneous fluctuations in tremor power in the absence of any task were tightly correlated with fluctuations in heart rate and pupil diameter (ie, 2 proxy measures of noradrenergic activity).^14^ Imaging and postmortem studies have shown that the integrity of the locus coeruleus (LC), the main cerebral source of noradrenaline, is associated with the presence or absence of PD tremor.^15^, ^16^ Although noradrenergic terminal function was decreased in PD patients versus controls, noradrenergic projections from the LC toward the thalamus were more preserved in patients with prominent tremor.^15^ Accordingly, lesioning noradrenergic LC terminals in a PD rat model reduced the development of tremor.^17^

In non‐PD tremor disorders, such as essential tremor, drugs acting on the noradrenergic system influence tremor and sympathomimetics increase tremor,^18^ whereas beta‐blockers reduce tremor.^19^ Some studies have suggested that beta‐blockers may improve PD tremor as well,^20^, ^21^, ^22^, ^23^, ^24^ but not all evidence points in the same direction^25^, ^26^ (Table S1). These studies most frequently administered propranolol, a non‐selective beta‐adrenergic receptor antagonist. Propranolol decreases the activity of the sympathetic nervous system, therefore, reducing the release of noradrenaline in the brain.^27^

Here, we used pharmacological imaging to investigate if and how propranolol influences PD tremor and to test if this role is specific to stressful conditions or not. We performed a placebo‐controlled, cross‐over trial to investigate the effect of propranolol on tremor severity (accelerometry) and tremor‐related activity (concurrent accelerometry‐functional magnetic resonance imaging [fMRI]). Based on previous findings,^6^ we hypothesized that propranolol would reduce resting tremor particularly during cognitive load, by inhibiting tremor‐related activity in the thalamus. We also tested the alternative hypothesis that the effects of propranolol on tremor generalize across stressful and resting conditions, given evidence for the role of noradrenergic mechanisms in PD tremor at rest.^14^

## Methods

### Study Population

We included 34 people with PD according to the Movement Disorders Society (MDS) criteria,^28^ with a prominent resting tremor in at least 1 arm (MDS Unified PD Rating Scale [MDS‐UPDRS] III tremor‐score ≥2; range 0–4). Exclusion criteria were: neurological or current psychiatric comorbidities; contraindications for MRI; cardiac arrhythmias; possible intolerance for beta‐blockers (bradycardia, peripheral vascular diseases, diabetes mellitus, chronic obstructive pulmonary disease, asthma, and hypotension); use of medication that may interact with propranolol or influences its metabolism; severe head tremor or dyskinesias; or cognitive impairment (Mini‐Mental State Examination [MMSE] score <26).^29^

Participants underwent an extensive phone screening before inclusion. Given the exploratory nature of this study and the lack of similar approaches, we did not perform a formal power calculation. Our desired sample size (n = 24 with MRI) was based on previous studies reporting the effects of (dopaminergic) pharmacological interventions on tremor‐related brain activity.^10^, ^30^

**FIGURE 1:**
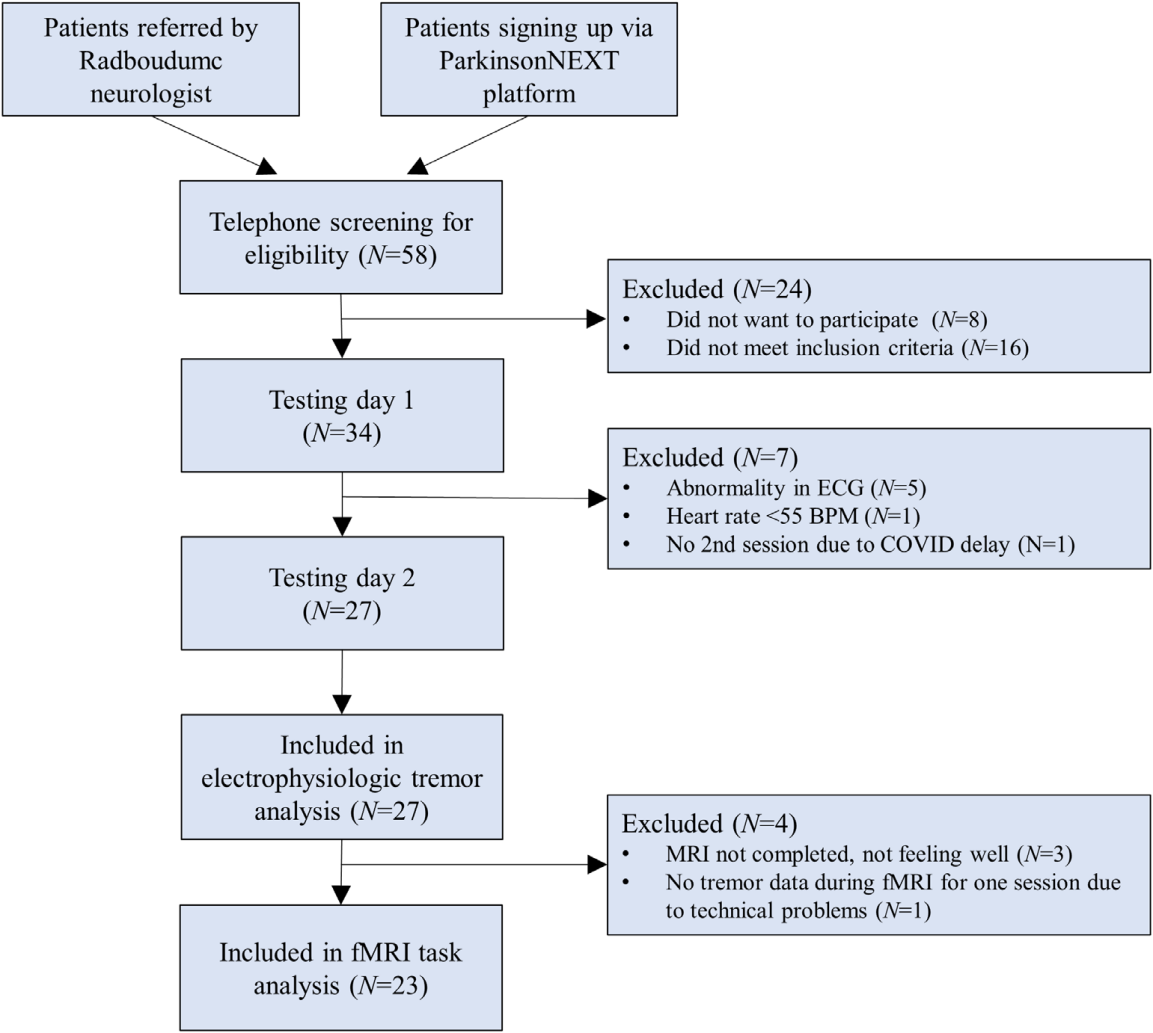
Participant flow chart. This participant flow chart shows how many participants were screened from the 2 recruitment sources, how many were eligible and how many were included per testing day and per analysis. [Color figure can be viewed at www.annalsofneurology.org]

Measurements took place at the Donders Center for Cognitive Neuroimaging in Nijmegen (March 2020–September 2021). We performed electrophysiological tremor assessments in 27 participants, of whom 24 underwent successful fMRI scanning during both sessions (1 patient excluded from fMRI analyses; final sample n = 23) (see Fig 1). Table 1 shows the characteristics of participants included in data analysis (n = 27).

**TABLE 1:** Clinical characteristics

Characteristic	Mean (±SD)/n
Sex	
F	9
M	18
Age (yr)	61.4 (8.2)
Disease duration (yr)	4.2 (2.9)
Most affected side	
Right	17
Left	10
Dopaminergic medication user	
Yes	26
No	1
LEDD (mg/day)	598.1 (386)
Mini Mental State Examination (total score)	28.8 (1.3)
HY stage	
HY 1	4
HY 2	22
HY 2.5	1
MDS‐UPDRS III (total score, OFF medication)	28.8 (10.8)
Tremor total	9.3 (4.4)
Resting tremor	6.1 (2.6)
Postural tremor	1.9 (1.4)
Kinetic tremor	1.2 (1.1)
Non‐tremor total	19.5 (8.2)
Bradykinesia and rigidity	13.7 (6.9)
Axial symptoms	5.9 (1.9)

Disease characteristics of participants (n = 27) included in the analysis of the electrophysiological tremor assessment. Some of these (n = 4) were not included in the fMRI‐analysis.

Axial symptoms = items 1–3 and 9–14; F = female; fMRI = functional magnetic resonance imaging; HY = Hoehn and Yahr; LEDD = levodopa equivalent daily dosage; M = male; MDS‐UPDRS = Movement Disorders Society Unified Parkinson's Disease Rating Scale; non‐tremor total = MDS‐UPDRS III items 1–14 (bradykinesia and rigidity = items 3 and 4–8; postural tremor = item 15; kinetic tremor = item 16); tremor total = MDS‐UPDRS III items 15–18 (resting tremor = items 17 + 18); SD = standard deviation.

### Ethical Approval and Patient Recruitment

Participant recruitment was performed through the Radboud University Medical Centre Neurology department and through patient recruitment platform ParkinsonNEXT (https://www.parkinsonnext.nl). All participants gave written informed consent before the experiment. The local medical ethical committee (METC Arnhem‐Nijmegen) approved all procedures and communication materials (reference 2016–3101; NL59724.091.16). The EudraCT number is 2016–004629‐18, and the study was also registered in the ISRCTN registry (ISRCTN89589002). This manuscript reports all items of the Consolidated Standards of Reporting Trials (CONSORT) checklist (randomized crossover‐trial extension).

### Experimental Design

**FIGURE 2:**
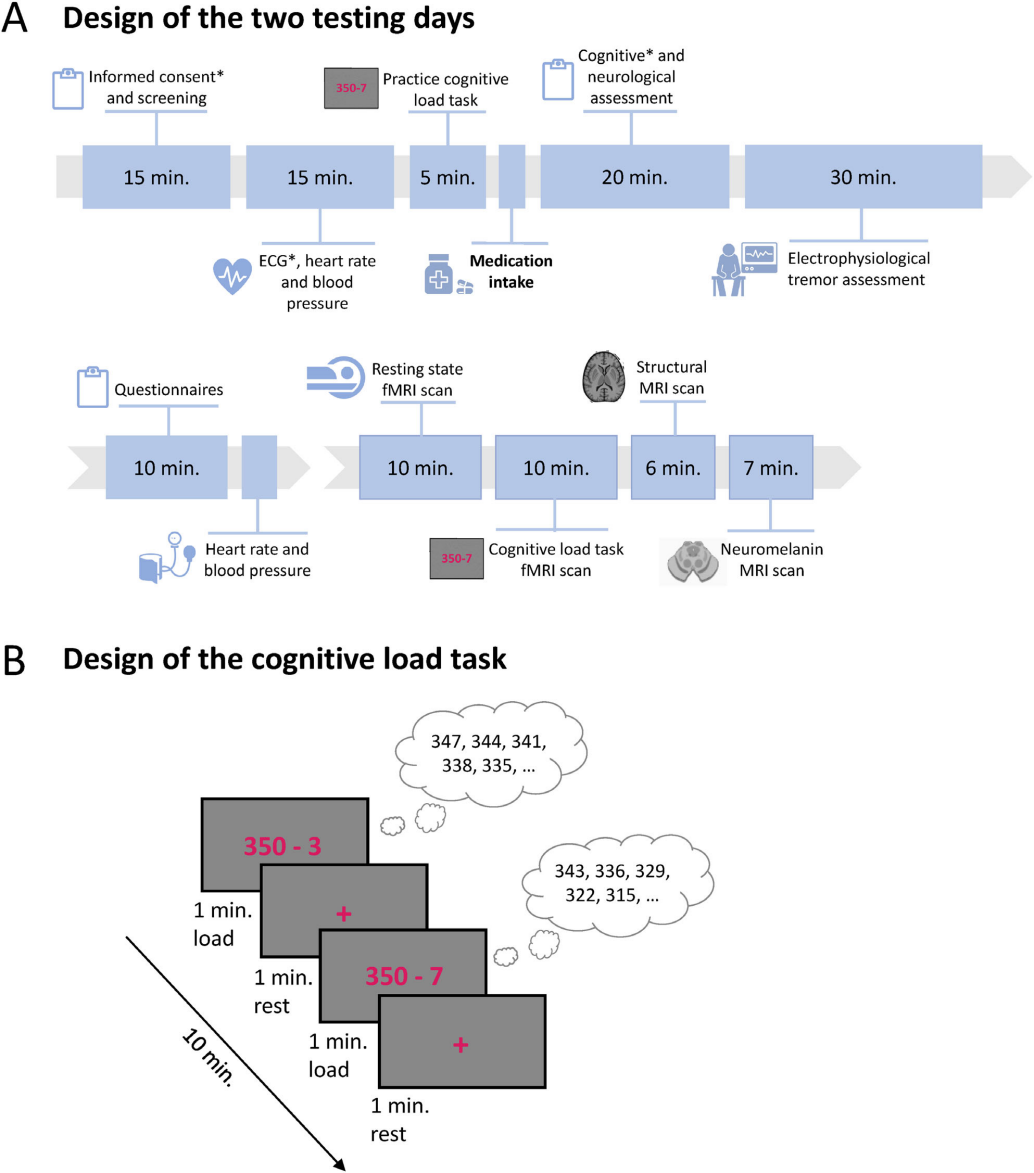
Study design and cognitive load task. (A) Shows an overview of the testing day. Each session consisted of a behavioral part and a part in the MRI scanner. (B) Visualizes the cognitive load task that was performed during fMRI scanning. *Only done during first testing day. ECG, electrocardiogram; fMRI, functional magnetic resonance imaging; MRI, magnetic resonance imaging. [Color figure can be viewed at www.annalsofneurology.org]

Our cross‐over, double‐blind, placebo‐controlled design consisted of 2 testing days of ~5 hours. Participants received propranolol (40 mg, dispersed in water) on 1 day, and a placebo (cellulose dispersed in water) on the other (order counterbalanced, Supplementary note S1). Each testing day consisted of clinical and behavioral tests and fMRI (Fig 2A).

At the start of the first visit, an electrocardiogram (ECG) was recorded and checked by a physician for irregularities in rhythm and conductance that could indicate contraindications for propranolol. Blood pressure and heart rate were monitored. Participants were in a practically defined OFF‐state during all measurements (>12 hours after the last dose of levodopa, >24 hours after dopamine agonists).

## Measurements

### General Clinical Measurements

During both sessions, motor symptoms were assessed using the MDS‐UPDRS III.^31^ Cognitive function was assessed during the first session with the MMSE.^32^

### Electrophysiological Tremor Assessment and Preprocessing

We performed an electrophysiological tremor assessment, with 4 conditions: rest (forearms on the armrests, hands unsupported), cognitive load (serial‐subtraction task), postural tremor (arms stretched forward), and kinetic tremor (slow wrist extension and flexion of the most‐affected arm). All conditions (each 1 minute) were repeated twice. Participants were told to never voluntarily suppress the tremor. MRI‐compatible electromyography (EMG)‐electrodes were placed on both forearm muscles (extensor digitorum communis and flexor carpi radialis), and, in case of leg tremor, on the tibialis anterior and gastrocnemius. An MRI‐compatible tri‐axial accelerometer (Brain Products, Gilching, Germany; sampling frequency [Fs] = 5,000Hz) was placed on the dorsum of the most‐affected hand.

Preprocessing involved detrending and demeaning the signal to remove temporal drifts and band‐pass filtering of 1 to 20Hz to filter out noise, as done before.^11^ We used Fieldtrip to calculate power spectra for each condition and each accelerometry channel (x/y/z) between 1 and 16Hz using a 5‐second Hanning taper, resulting in a 0.2Hz spectral resolution. Only fluctuations within the frequency ranges of 3.5 to 7Hz for resting tremor (rest and cognitive load conditions) and 3.5 to 10Hz for postural and kinetic tremor were considered as tremor‐related activity. The log10‐transformed peak power was determined for the channel with the highest power.

### MRI Image Acquisition and Preprocessing

fMRI was performed on a Siemens PRISMA 3 T MRI‐system with a 32‐channel head–neck coil. We used a multi‐band echo planar imaging sequence with multi‐band acceleration factor 6, repetition time 1,000ms, echo time 34ms, 72 axial slices, 2.0mm isotropic voxels and field of view 210mm (~10 minutes, 600 images). Anatomical images were acquired using a magnetization‐prepared rapid gradient‐echo sequence, repetition time 2,300ms, echo time 3.03ms, 192 sagittal slices, 1.0mm isotropic voxels, and field of view 256mm (~5 minutes). Participants were instructed to lie still with their eyes open (confirmed with online eye‐tracking).

Preprocessing of functional and structural images was done using fMRIprep v20.2.1.^33^ Preprocessing steps for structural scans involved intensity nonuniformity correction, skull‐stripping, brain surface reconstruction, spatial normalization to Montreal Neurological Institute (MNI) space, and brain tissue segmentation. Preprocessing for the functional runs included correction for motion‐related variance using independent component analysis‐automatic removal of motion artifacts (ICA‐AROMA), nuisance regression of average cerebrospinal fluid and white matter time series together with 24 motion derivatives, and high‐pass filtering (>0.007Hz). ICA‐AROMA components were visually checked by 2 researchers and manually corrected if necessary. The first 5 images of functional runs were discarded. The functional scans were co‐registered to the T1‐weighted reference image and mapped to MNI space. Preprocessed functional images were spatially smoothed using a Gaussian kernel of 6mm full width at half‐maximum using SPM12 (https://www.fil.ion.ucl.ac.uk/spm/software/spm12).

### fMRI Task: Cognitive Load Task

Participants performed a validated 10‐minute stress‐inducing cognitive load task during both sessions (propranolol and placebo). This task consisted of alternating 1‐minute blocks of mental arithmetic (cognitive load) and observation of a cross (rest) (see Fig 2B).^6^ Participants were instructed to perform the mental calculations (in silence) as fast as possible and to start again if they reached 0. We used continuous eye‐tracking of the left eye (Eyelink 1,000 plus, Fs = 1,000Hz) to monitor pupil diameter, we measured heart rate using a pulse oximeter on the thumb of the less‐affected hand (Blood Pulse Sensor; Brain Products), and we continuously measured tremor during the task as outlined below.^6^, ^12^ After the task, participants rated their perceived stress level, separately for cognitive load and rest blocks, using a visual analog scale (range, 1–5).

### Debriefing

After the final testing day, all participants were asked which medication they thought they had received that day.

## Statistical Analysis

We tested for effects of propranolol versus placebo (DRUG), cognitive load versus rest (BLOCK), and their interaction, on (1) tremor‐related brain activity (BOLD; primary outcome); (2) tremor power (accelerometry or EMG, both inside and outside the scanner; co‐primary outcome), (3) stress measures (pupil diameter, heart rate and perceived stress; secondary outcome). We tested the hypothesis that propranolol reduces tremor‐related activity and tremor power specifically during stress (BLOCK*DRUG interaction). Analyses were performed using R‐4.2.1 (www.r-project.org).

### Tremor Power

Resting tremor power was calculated at each individual's tremor peak, log10‐transformed, and averaged across repetitions. For postural and kinetic tremor, we performed separate 1‐sided paired *t* tests comparing the effect of DRUG on tremor peak power. We did the same analyses with tremor frequency as dependent variable. For 1 participant, only tremor frequency was compared between sessions, and tremor power could not be computed because of technical issues. Inside the fMRI scanner, accelerometry data were unavailable in 3 participants because of technical issues.

### Stress Measures

We performed 2‐way repeated measures analysis of variance (rm‐ANOVA) to test the effects of BLOCK and DRUG on heart rate, pupil diameter, and perceived stress level. Pupil size data during fMRI were of insufficient quality in 1 or both sessions for 10 participants, because of dropping of the eye lid, insufficient light, or inability to visualize the pupil. Heart rate data were of insufficient quality in 9 participants.

### Tremor‐Related Brain Activity (fMRI)

At the first (subject‐specific) level, we performed a multiple regression analysis in SPM12 with separate regressors modeling rest and cognitive load conditions for each session. As done before, we modelled tremor‐related activity by adding scan‐by‐scan tremor power (accelerometry) and its first temporal derivative as 2 parametric modulation regressors for each of these 4 conditions.^6^ To remove non‐neural and motion‐related noise, we included covariates of no interest (calculated by fMRIprep^33^) for framewise displacement, standardized derivative of root‐mean‐square variance over voxels (DVARS), cerebrospinal fluid, white matter signal, 24 head‐motion derivatives, and ICA‐AROMA components to the first‐level contrasts. For each individual, this resulted in 4 contrast‐images depicting tremor‐related activity for each condition (rest vs cognitive load; propranolol vs placebo), and in separate contrast images depicting task‐related activity (cognitive load > rest; propranolol vs placebo). For participants with left as most‐affected hand (n = 9), we flipped the contrast images in the axial plane for tremor power‐related and tremor change‐related contrasts.

At the second (group) level, we first verified that individuals showed task‐related activity in a cognitive control network (cognitive load > rest, averaged across medication sessions) and tremor‐related activity in the cerebello‐thalamo‐cortical circuit (averaged across blocks and medication sessions), as done before.^6^ We used threshold free cluster enhancement (TFCE) in SPM12 (http://dbm.neuro.uni-jena.de/tfce/) and assessed significance with non‐parametric permutation testing (10,000 permutations). We corrected for multiple comparisons (threshold *p* < 0.05), familywise error (FWE) corrected at the voxel‐level for all analyses. Corrections were done across the whole brain for task‐related activity and within regions of interest (ROI) (Table S2) in the cerebello‐thalamo‐cortical circuit for tremor‐related activity, given previous work.^13^ Mean framewise displacement for each participant and session was added as covariate. We used the SPM Anatomy Toolbox 2.1 for anatomical localization of activity clusters.^34^

Second, we used rm‐ANOVAs to test for effects of BLOCK and DRUG on tremor‐related activity within the cerebello‐thalamo‐cortical circuit (average beta‐values depicting tremor‐related activity in each ROI). We also performed an exploratory whole brain search.

Finally, we assessed the relationship between drug‐related changes in tremor power and drug‐related changes in tremor‐related brain activity with Spearman correlations (R‐4.2.1). For this, we calculated the change in mean beta‐values for each ROI showing effects of propranolol and the change in tremor power, per participant.

We also report Bayesian statistics to test the validity of null responses, particularly regarding our main hypothesis (BLOCK*DRUG interaction on tremor‐related brain activity and resting tremor power). Bayes factors (BF_10_) of 1 to 3, 3 to 10, or >10 were, respectively, considered anecdotal, moderate, or strong evidence for the alternative hypothesis, whereas BF_10_ of 0.33 to 1, 0.1 to 0.33, or <0.1 were considered anecdotal, moderate, or strong evidence for the null hypothesis (Supplementary S1 note 2).

## Results

### Tremor Power

**FIGURE 3:**
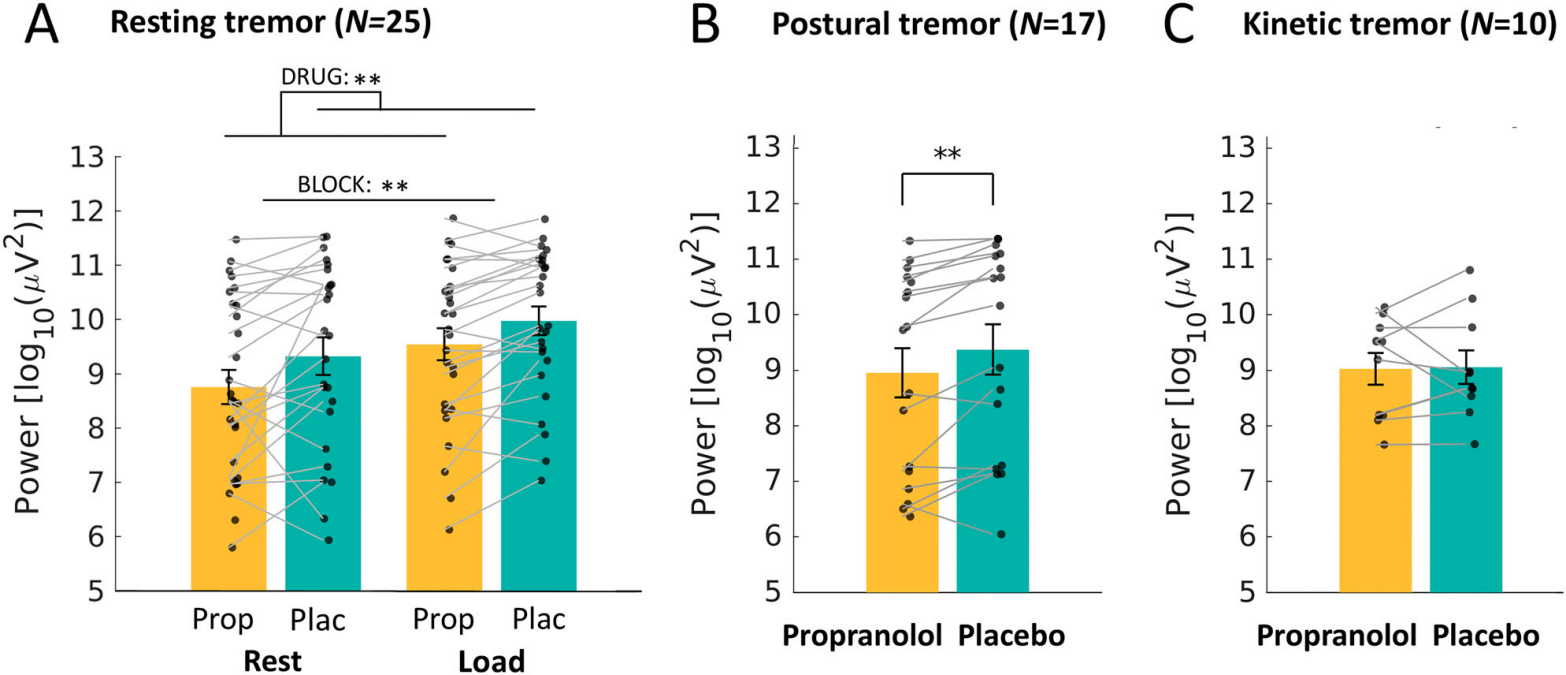
Effect of propranolol on different types of Parkinson's disease tremor. The effect of propranolol on average tremor power (±standard error of the mean), measured with accelerometry during two 1‐minute blocks per condition, for (A) resting tremor, (B) postural tremor and (C) kinetic (action) tremor. *p < 0.05; **p < 0.01; ***p < 0.001. [Color figure can be viewed at www.annalsofneurology.org]

Outside the scanner, cognitive load increased rest tremor power (main effect BLOCK: *F*[1,24] = 10.5; *p* = 0.003; $\eta_{p}^{2}$ = 0.31), whereas propranolol reduced rest tremor power (main effect DRUG: *F*[1,24] = 10.6; *p* = 0.003; $\eta_{p}^{2}$ = 0.31) (Fig 3A). We found no BLOCK*DRUG interaction (*F*[1,24] = 0.0; *p* = 0.95; $\eta_{p}^{2}$ = 0.00; BF_10_ = 0.27). Rest tremor frequency was unaffected by propranolol (main effect DRUG: *F*[1,25] = 0.7; *p* = 0.42; $\eta_{p}^{2}$ = 0.03), or cognitive load (main effect BLOCK: *F*[1,25] = 3.0; *p* = 0.10; $\eta_{p}^{2}$ = 0.11); we found no BLOCK*DRUG interaction (*F*[1,25] = 0.1; *p* = 0.74; $\eta_{p}^{2}$ = 0.00). Propranolol also reduced postural tremor power (*t* [16] = 3.6; *p* = 0.003; Cohen's d = 0.86) (see Fig 3B), but not its frequency (*t* [17] = 1.1; *p* = 0.28; Cohen's d = −0.27). Kinetic tremor was present across both sessions in only 10 participants. Propranolol did not affect tremor power (*t* [9] = 0.1; *p* = 0.89; Cohen's d = 0.05) (see Fig 3C) or frequency (*t* [9] = 0.7; *p* = 0.51; Cohen's d = 0.22).

**FIGURE 4:**
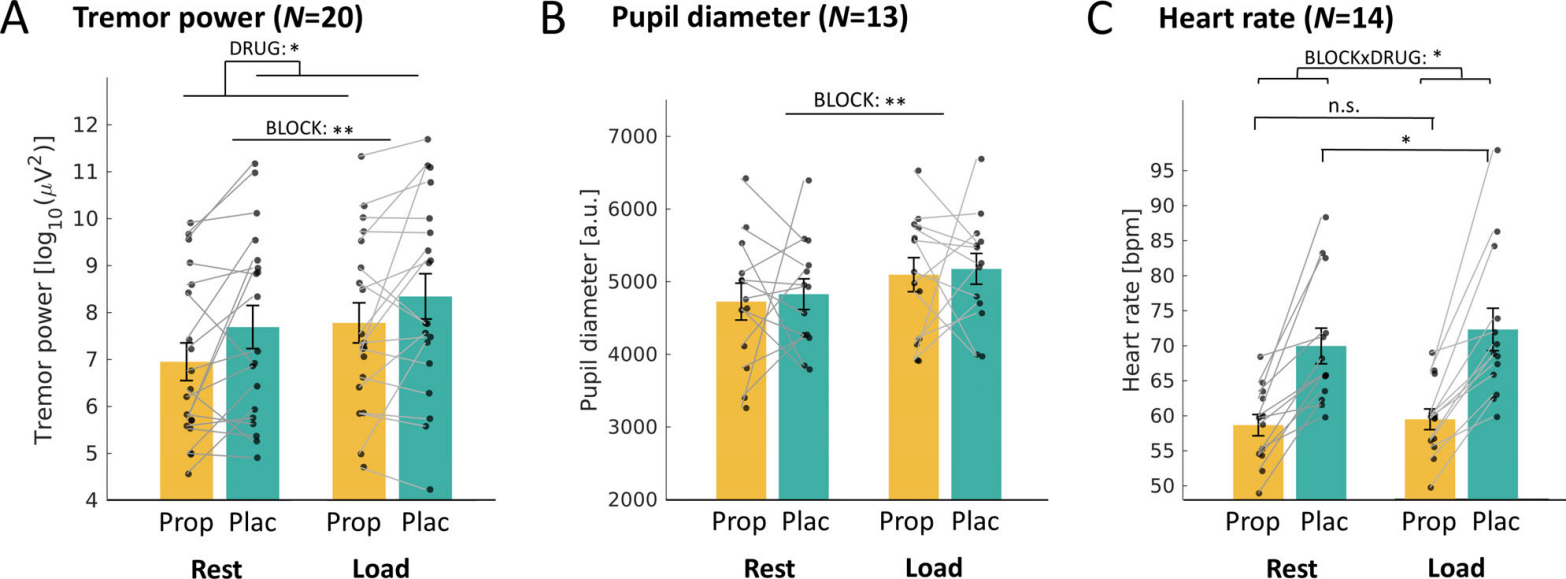
Effects of propranolol and cognitive load on physiological measures. The effect of cognitive load and propranolol intake on (A) tremor power, (B) pupil diameter, and (C) heart rate during the functional magnetic resonance imaging cognitive load task. *p < 0.05; **p < 0.01; ***p < 0.001. a.u, arbitrary unit; bpm, beats per minute. [Color figure can be viewed at www.annalsofneurology.org]

During MRI, we observed the same effects as outlined above. Cognitive load increased rest tremor power (main effect BLOCK: *F*[1,19] = 13.8; *p* = 0.001; $\eta_{p}^{2}$ = 0.42), whereas propranolol reduced tremor power (main effect DRUG: *F*[1,19] = 6.4; *p* = 0.02; $\eta_{p}^{2}$ = 0.25) (Fig 4A). There was no BLOCK*DRUG interaction (*F*[1,19] = 0.7; *p* = 0.41; $\eta_{p}^{2}$ = 0.04; BF_10_ = 0.50).

### Stress Measures

Inside the scanner, pupil size increased during cognitive load (main effect BLOCK: *F*[1,12] = 19.2; *p* = 0.001; $\eta_{p}^{2}$ = 0.62), but there was no effect of propranolol (main effect DRUG: *F*[1,12] = 0.1; *p* = 0.78; $\eta_{p}^{2}$ = 0.01) (see Fig 4B) and no interaction (*F*[1,12] = 0.0; *p* = 0.85; $\eta_{p}^{2}$ = 0.00; BF_10_ = 0.41). For heart rate, there was a significant BLOCK*DRUG interaction (*F*[1,13] = 5.3; *p* = 0.04; $\eta_{p}^{2}$ = 0.29). Post hoc tests showed that heart rate increased for cognitive load compared to rest during the placebo session (*t* [13] = 2.8; *p* = 0.02; Cohen's d = 0.75), but not during the propranolol session (*t* [13] = 1.1; *p* = 0.29; Cohen's d = 0.30) (see Fig 4C). Participants perceived the cognitive load blocks as more stressful than rest blocks (average score 2.7 vs 2.0 [range 1–5]; main effect BLOCK: *F*[1,22] = 19.4; *p* = 0.000; $\eta_{p}^{2}$ = 0.47). They perceived more stress during the placebo session (average score 2.6 vs 2.0; main effect DRUG: *F*[1,22] = 6.7; *p* = 0.02; $\eta_{p}^{2}$ = 0.23). There was no interaction (*F*[1,22] = 0.6; *p* = 0.44; $\eta_{p}^{2}$ = 0.03).

### Effects of Cognitive Load on Brain Activity

**FIGURE 5:**
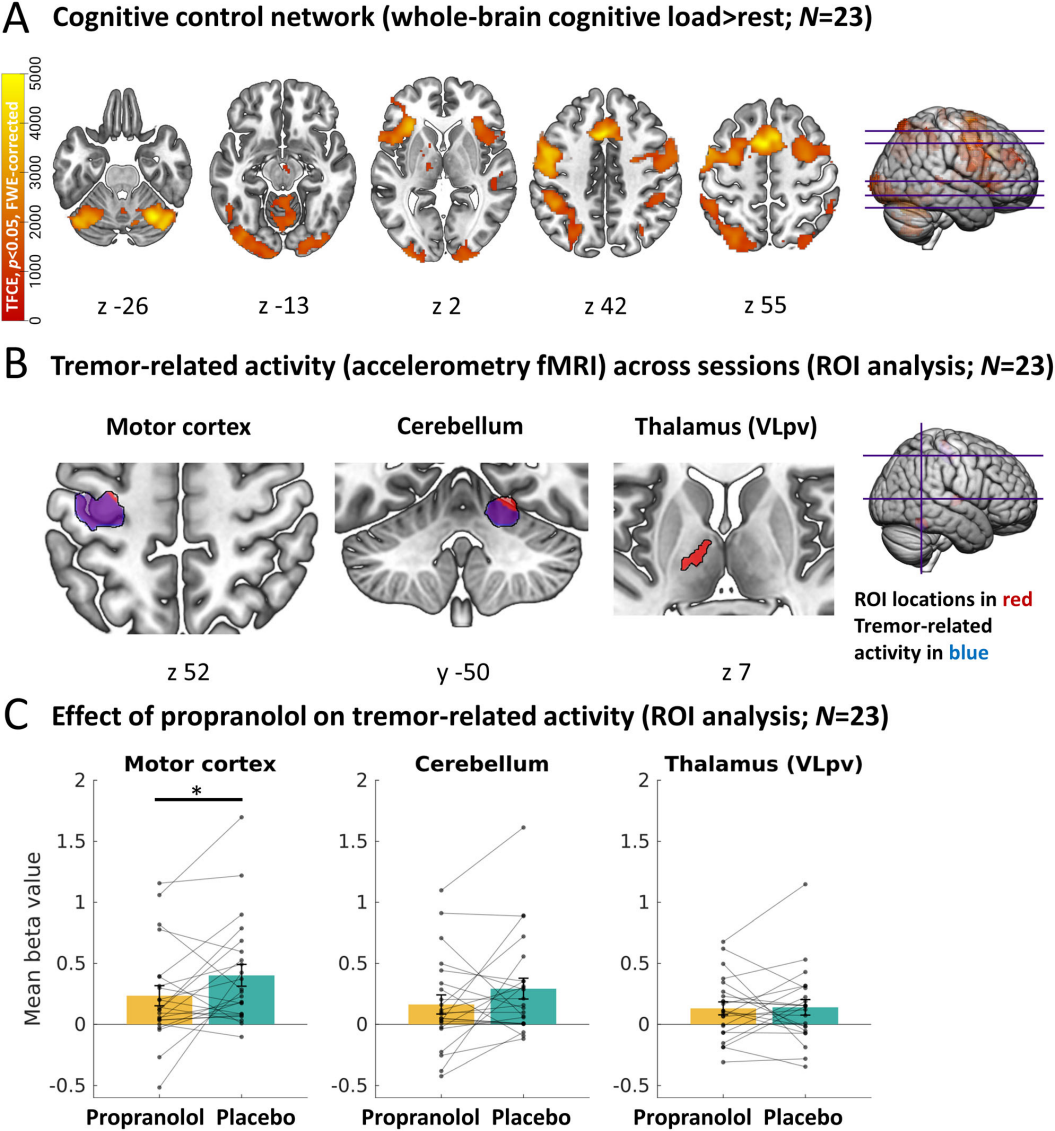
Cerebral activity patterns measured by functional magnetic resonance imaging (fMRI). (A) shows the activation of a cognitive control network during the cognitive load task (whole‐brain cognitive load > rest, averaged across sessions). The image shows threshold free cluster enhancement (TFCE)‐values of significant clusters, familywise error (FWE)‐corrected. (B) shows tremor power‐related activity across blocks in the cerebello‐thalamo‐cortical network (region of intrest [ROI]‐based analysis; averaged across sessions and blocks). The image shows significant clusters, FWE‐corrected. (C) shows the comparison of tremor‐related activity during the placebo and propranolol session, averaged over significant voxels in the cerebello‐thalamo‐cortical network (ROI‐based analysis; averaged across blocks). Bars represent mean beta values (±standard error of the mean) and dots show individual averaged beta values. *p < 0.05; **p < 0.01; ***p < 0.001. VLpv, ventrolateral nucleus of the thalamus, pars ventralis. [Color figure can be viewed at www.annalsofneurology.org]

Cognitive load was associated with increased activity in a cognitive control network, including the posterior‐medial frontal cortex, middle frontal cortex, superior and inferior parietal lobule, superior temporal lobe, and cerebellum (Fig 5A) (anatomical details in Table S3). Propranolol had no effect on task‐related activity, and there was no BLOCK*DRUG interaction.

### Tremor‐Related Brain Activity

Across sessions and conditions, we replicated previous findings showing tremor power‐related activity within the contralateral motor cortex, ipsilateral cerebellum, and contralateral VLpv (ventrolateral nucleus of the thalamus, pars ventralis; the latter trend‐significant) (see Fig 5B). Anatomically, these clusters largely overlapped with clusters found in previous studies.^6^, ^13^ We did not observe tremor change‐related activity in the basal ganglia, similar to a previous study using the same task^6^ (Table S3).

Propranolol significantly reduced tremor‐related activity in the motor cortex independent of cognitive load (main effect DRUG: *F*[1,21] = 5.3; *p* = 0.03; $\eta_{p}^{2}$ = 0.20) (see Fig 5C). In the cerebellum, the effect of propranolol approached significance (main effect DRUG: *F*[1,21] = 3.1; *p* = 0.09; $\eta_{p}^{2}$ = 0.13). Propranolol did not influence tremor‐related activity in the thalamus (VLpv; main effect DRUG: *F* [1, 21] =0.0; *p* = 0.93; $\eta_{p}^{2}$ = 0.00). There were no main effects of BLOCK on tremor‐related activity, and BLOCK*DRUG interactions were not significant (motor cortex: *F*[1,22] = 1.3; *p* = 0.26; $\eta_{p}^{2}$ = 0.06; BF_10_ = 0.50; cerebellum: *F*[1,22] = 0.2; *p* = 0.67; $\eta_{p}^{2}$ = 0.01; BF_10_ = 0.26; VLpv: *F*[1,22] = 0.3; *p* = 0.57; $\eta_{p}^{2}$ = 0.01; BF_10_ = 0.38).

Whole‐brain analyses showed tremor‐related activity in the contralateral motor cortex (peak voxel MNI [−33, −29, 56]; cluster size 402; *p* < 0.001 FWE‐corrected) and the ipsilateral cerebellum (peak voxel MNI [5, −51, −10]; cluster size 146; *p* = 0.001 FWE‐corrected) across sessions, but did not reveal tremor‐related BLOCK or DRUG effects in regions outside the cerebello‐thalamo‐cortical circuit.

There was no correlation between session‐specific changes (placebo > propranolol) in tremor power (accelerometry) and tremor‐related brain activity in the motor cortex (fMRI): rho = 0.12; *p* = 0.63.

### Debriefing

After completion of the second testing day, 19 of 27 participants guessed correctly in which order they had received the medication.

## Discussion

We investigated the effect of propranolol on both tremor power (accelerometry) and tremor‐related brain activity (fMRI) in 27 people with PD resting tremor. Based on previous findings,^6^ we hypothesized that propranolol would inhibit the stress‐related increase in tremor power and tremor‐related activity. There are 2 main findings. First, cognitive load increased tremor power, whereas propranolol reduced tremor power, independently of the task (no interaction). This supports the hypothesis that PD tremor is modulated by noradrenergic activity and not only during stressful conditions, but also during “rest.” Second, propranolol reduced tremor amplitude‐related activity in the contralateral motor cortex, but not in the ipsilateral cerebellum or the thalamic VLpv nucleus.

### Effects of Propranolol on Tremor Power

Propranolol reduced tremor power across 2 separate measurements in different contexts (ie, outside the scanner [seated] and inside the scanner [lying supine]) suggesting this finding is robust. Our findings also suggest that the effect of propranolol on PD tremor is relevant. The magnitude of tremor reduction after propranolol was similar to the increase observed from rest to cognitive load blocks ($\eta_{p}^{2}$ effect‐size of 0.31 for both effects). This worsening of tremor during cognitive load is a common and clearly visible observation in clinical practice, experienced as impactful by patients.^35^ In previous work, cognitive load increased the UPDRS tremor‐item by 1 full point (scale 0–4).^7^ Although there are no validated clinically meaningful differences for tremor accelerometry, this indirectly suggests that the effect of propranolol is clinically relevant.

The tremor‐reducing effect of propranolol was not specific to the stressful (cognitive load) condition, but was also effective during rest periods. These findings are in line with a previous study in 7 PD patients where propranolol counteracted the tremor‐increase caused by adrenaline injection, without impact on tremor‐increase observed during mental calculations.^36^ Effects of propranolol on tremor at rest were not assessed in that work. Our findings cannot be attributed to the cognitive load task failing to activate the noradrenergic stress system, because we observed clear task effects on proxies of (nor)adrenergic activity (ie, increased heart rate [in the placebo session] and pupil diameter) (see Fig 4). Additionally, we observed task‐related activity in a cognitive control network previously linked to psychological stress (see Fig 5A).^37^ It is also unlikely that our study was underpowered to detect a BLOCK*DRUG interaction, and Bayesian statistics convincingly supported the null hypothesis (BF <0.3). Instead, our findings suggest a more general effect of propranolol on PD tremor, beyond stressful situations. This fits with recent findings showing that spontaneous tremor power fluctuations at rest correlate with fluctuations in proxies for noradrenergic activity (pupil and heart rate).^14^ The clinical implication of our findings is that propranolol could be a useful additional treatment option for PD tremor, whether or not it is triggered by stress, particularly for tremor responding insufficiently to dopaminergic drugs.

Propranolol effectively reduced both resting tremor and postural tremor, but not kinetic tremor. This suggests that the noradrenergic system plays no role in kinetic tremor, although the small sample number of patients with kinetic tremor (n = 10) warrants caution when interpreting this result (Supplementary note 3).

### Propranolol Inhibits Tremor‐Related Brain Activity

Tremor was associated with a cerebral circuit comprising the contralateral motor cortex, ipsilateral cerebellum, and contralateral thalamic VLpv nucleus (exhibiting a trend toward significance). Propranolol significantly reduced tremor‐related activity in the motor cortex, but not in the cerebellum (which was trend‐significant) or the VLpv. Similar to the effects we observed on tremor power, but in contrast to our hypothesis, there was no BLOCK*DRUG interaction in any of these 3 regions. Bayesian evidence in favor of the null hypothesis was anecdotal to moderate for all regions (BF_10_ = 0.38–0.50), and with very small effect sizes (0.0–0.1). It is unlikely that studies in larger samples would detect a clinically relevant interaction effect.

Our finding that propranolol reduced tremor‐related activity in the motor cortex, independent of the cognitive task, fits with the notion that the LC has anatomical projections to all nodes of the cerebello‐thalamo‐cortical circuit.^38^ Furthermore, the motor cortex, with a particularly high glucocorticoid receptor density, is susceptible to the effects of stress.^39^ In animal studies, application of noradrenergic antagonists over the primary motor cortex hyperpolarized layer‐V output neurons and decreased firing rates,^40^ which is in line with our findings.

Importantly, stress‐related noradrenergic activity activates several receptors (including beta‐1, beta‐2, beta‐3, and alpha‐receptors), whereas propranolol only blocks beta‐1 and beta‐2 receptors.^27^ The latter are differently expressed within the cerebello‐thalamo‐cortical circuit (Supplementary Fig S1). Assessing the genetic expression of these receptors, derived from postmortem samples (Allen Brain Institute^41^; MNI‐spatial maps at https://neurosynth.org/genes), showed a high genetic expression of beta‐1 receptors in the motor cortex ROI (z = 0.32) that is not seen in the thalamic VLpv nucleus (z = −0.41) or cerebellum (z = −0.08). Moreover, the genetic expression of beta‐2‐receptors is found mainly in the VLpv (z = 0.70), but not in the motor cortex (z = −0.22) or cerebellum (z = −0.45). Our finding that propranolol reduced tremor‐related activity only in the motor cortex suggests that beta‐1 receptors were primarily targeted, but further research should confirm this.

### Peripheral Contributions

We cannot rule out that some of the clinical effects are explained by peripheral effects of propranolol. Although PD tremor is primarily generated by cerebral oscillations, it is well‐accepted that peripheral mechanisms play an additional role. For example, PD tremor can be influenced by somatosensory afferents, because changes in tremor power may occur even after slight adaptations in limb posture.^42^ However, studies showed that mechanical perturbations could only reset PD tremor in a subsample of participants^43^ or only in specific conditions.^44^ Somatosensory afferents might also have a role in stabilizing the tremor rhythm, as was substantiated by a study showing that deafferentation of a tremulous arm did not affect tremor power or frequency.^45^ Because propranolol has both peripheral and central effects, it may reduce tremor through a combined action on both.^46^ Because the extent of peripheral contributions to PD tremor may vary between people, the effect of propranolol on tremor via the cerebello–thalamo–cortical circuit might differ as well. This may explain why we did not find a correlation between propranolol‐induced tremor power reduction and the effect of propranolol on the motor cortex.

### Limitations

The main strength of our study is that our multi‐modal design allowed us to address both the clinical effect of propranolol on tremor and the underlying mechanisms. Some limitations should be considered. First, our sample is modest, and drop‐out was relatively high (32%), mostly because of stringent ECG‐based exclusion criteria. Second, although the study was double‐blind, 19 of 27 participants guessed correctly in which order they had received the medication. Unblinding may have occurred because of people noticing the effects of propranolol on heart rate (that decreased by 12.4 bpm) or tremor itself. Third, although our results suggest that propranolol did not affect tremor‐related thalamic (VLpv) activity, Bayesian analyses could not rule out this effect completely, possibly because our fMRI multi‐band scanning protocol is less sensitive to activity in subcortical regions, including the thalamus.^47^

### Clinical Implications

This placebo‐controlled study shows that propranolol is effective in reducing PD tremor across both stressful and resting conditions. Insights from this study may improve treatments that attenuate the influence of the stress system on PD tremor, based on behavioral (eg, mindfulness‐based interventions)^8^, ^48^ or pharmacological suppression (eg, propranolol). Our findings raise the possibility that the effect of propranolol is mediated by beta‐1 receptors in the motor cortex, which—if confirmed—opens opportunities for more targeted interventions with fewer side effects than propranolol. Furthermore, because noradrenaline has been shown to exert long‐term effects on the motor cortex by promoting long‐term plasticity,^49^ daily use of propranolol use may have beneficial effects that extend beyond the single‐dose findings we report.

## Author Contributions

R.H. and B.B. contributed to the conception and design of the study; A.H., M.W., D.P., D.G., T.P., F.G., and M.D. contributed to the acquisition and analysis of data; A.H. and R.H. contributed to drafting the text or preparing the figures.

## Potential Conflicts of Interest

Nothing to report.

## Supporting information


**Data S1.** Supporting Information.

## Data Availability

All derived and anonymized individual data supporting these findings are openly available at the Donders Repository at https://data.ru.nl/collections/di/dccn/DSC_3024005.02_550. All scripts used to generate the analyses presented in this paper are publicly archived at https://github.com/AnoukvanderHeide/Propranolol_PD_tremor.
